# Surface strain measurements of fingertip skin under shearing

**DOI:** 10.1098/rsif.2015.0874

**Published:** 2016-02

**Authors:** Benoit Delhaye, Allan Barrea, Benoni B. Edin, Philippe Lefèvre, Jean-Louis Thonnard

**Affiliations:** 1 Institute of Neuroscience (IoNS), Université catholique de Louvain, Brussels and Louvain-la-Neuve, Belgium; 2 Institute of Information and Communication Technologies, Electronics and Applied Mathematics (ICTEAM), Université catholique de Louvain, Brussels and Louvain-la-Neuve, Belgium; 3 Cliniques Universitaires Saint-Luc, Physical and Rehabilitation Medicine Department, Université catholique de Louvain, Brussels and Louvain-la-Neuve, Belgium; 4 Department of Organismal Biology and Anatomy, University of Chicago, Chicago, IL, USA; 5 Physiology Section, Department of Integrative Medical Biology, Umeå University, Umeå, Sweden

**Keywords:** skin mechanics, friction, touch, tactile perception

## Abstract

The temporal evolution of surface strain, resulting from a combination of normal and tangential loading forces on the fingerpad, was calculated from high-resolution images. A customized robotic device loaded the fingertip with varying normal force, tangential direction and tangential speed. We observed strain waves that propagated from the periphery to the centre of the contact area. Consequently, different regions of the contact area were subject to varying degrees of compression, stretch and shear. The spatial distribution of both the strains and the strain energy densities depended on the stimulus direction. Additionally, the strains varied with the normal force level and were substantial, e.g. peak strains of 50% with a normal force of 5 N, i.e. at force levels well within the range of common dexterous manipulation tasks. While these observations were consistent with some theoretical predictions from contact mechanics, we also observed substantial deviations as expected given the complex geometry and mechanics of fingertips. Specifically, from in-depth analyses, we conclude that some of these deviations depend on local fingerprint patterns. Our data provide useful information for models of tactile afferent responses and background for the design of novel haptic interfaces.

## 1. Introduction

How the complex interactions between human skin and external objects are translated to tactile information remains to a large extent an enigma. It is clear, however, that these interactions result in specific spatio-temporal patterns of strain in the skin and subjacent tissues that depend on the mechanical properties of both the object and the fingertip. The tactile mechanoreceptors embedded in the fingertips respond to various aspects of these stresses and strains with action potentials [1–4]. The resulting afferent signals ultimately allow the brain to extract high-level features of the object (e.g. shape, texture and weight) in a context-dependent manner, or to trigger-specific actions (e.g. grip force adjustments). Understanding the biomechanics of both the skin and subcutaneous tissue is therefore fundamental for our understanding of the human tactile sensory system.

Several attempts have been made to measure the stresses and strains in the skin resulting from a given stimulus, and to model skin properties using these measurements. Most of these studies focused on normal loading of the finger by points, lines or flat loading surfaces. These studies show that the geometry of the fingertip has a profound effect on stress distribution and intensity [5]. While reasonable predictions of skin deflection under different indentation profiles have been obtained with homogeneous elastic models, even higher accuracy has resulted from finite-element model simulations (FEM) based on multi-layered skin [6–9] and models based on incompressible fluid-filled membranes [10–12]. The pressure distribution measured under normal indentation showed asymmetric distribution profiles again underlining the importance of the complex geometry of subcutaneous tissues for its responses to mechanical loading [13,14]. Furthermore, tangential stresses occur in the fingertip in response to fully normal (non-tangential) loads, with amplitudes that depend on surface friction [15].

Measured and modelled deformations have been related to afferent responses recorded in primate peripheral nerves. The afferent responses of SA-1 afferents in response to normally loaded bars and edges could be precisely predicted based on maximal compressive strain and local strain energy density [1,2,16,17]. Importantly, however, these models accounted for static stimuli; aspects of the dynamics might be better predicted by FA-1 afferents. Given the complex geometry and continuum mechanics of the fingertip, the stresses and strains that the different types of mechanoreceptors are subjected to are considerably impacted by their location within the skin. Interestingly, FEM studies indicate that stresses are concentrated at fingerprints and papillae ridges, the very sites where mechanoreceptors are preferentially located [18,19]. Much less is known, however, about the skin mechanics under simultaneous normal and tangential loading, even though everyday manipulation tasks (e.g. grip and explorative touch) nearly always include a tangential loading component (e.g. object weight, static or sliding frictional forces). The fingertip appears to be viscoelastic with respect to tangential stresses and shows increasing stiffness with increasing strain amplitude [20–23]. Individual afferents responding to tangential force stimuli show unique directional sensitivity profiles [24]. Given the complex structure of the fingertip, FEM analyses of its biomechanical properties require a large number of free parameters to accurately describe the geometry and the mechanics with high fidelity (e.g. thickness and elasticity of the different layers, fingerprint geometry, etc.). It is therefore very important to fit these models with precise data.

We recently showed that increasing the tangential force between a surface and the finger (similar to that present during object lifting) creates partial slips in the contact area that precede full slip of the object [25,26]. These partial slips first occur at the periphery of the contact area, and then propagate to the centre of contact. They are associated with progressively reduced stable contact and are tuned to the direction of stimulation [27]. These findings, that some parts of the skin are in stable contact with the surface while other parts are sliding, necessarily imply that surface strains take place within the contact area. In this study, we measure these strains by imaging the contact between the fingerpad and a smooth transparent glass surface while applying various normal and tangential loads that induce sliding in four directions (proximal, distal, radial and ulnar). Green–Lagrange strains were then derived with high spatio-temporal resolution from the displacement field obtained using computer vision techniques.

We observe highly patterned and reproducible strain waves coming from the periphery of the contact area and propagating towards its centre. These observations contrast with theoretical predictions based on contact mechanics theory. Our results provide information for the design of more precise fingertip models and novel haptic interfaces. Furthermore, they will inform peripheral afferent models that predict responses to tangential loading.

## 2. Methods

### 2.1. Participants and data collection

Eight healthy volunteers participated in the study (eight males, aged 23–29) after giving informed consent. The local ethics committee approved the study.

**Figure 1.**
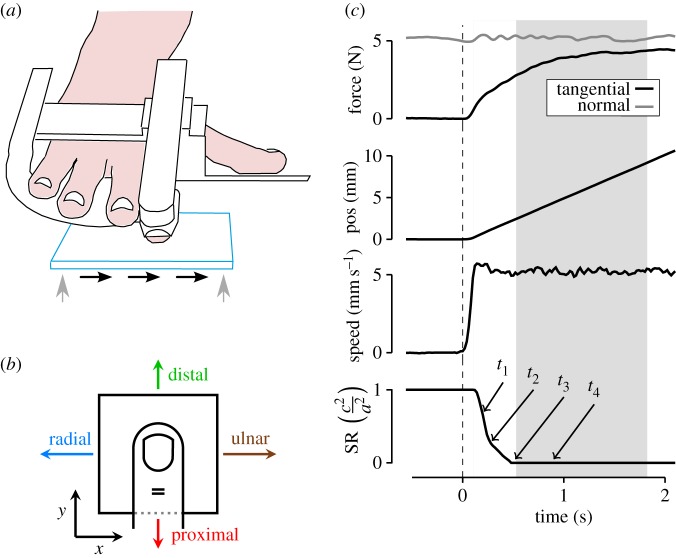
Experimental apparatus, procedures and data analysis. (*a*) Experimental apparatus. The subject's hand rested in the hand support, with the right index finger fixed. The horizontal glass plate moved by means of a robot actuator. The plate loaded on the finger (servoed normal force) and moved sufficiently far such that the finger was completely sliding in one of four directions with position control. (*b*) Stimulus directions: directions correspond to the movement of the glass plate relative to the fixed finger. ‘Radial’ is towards the thumb side of the hand and ‘proximal’ is towards the wrist. (*c*) Forces, position and speed profiles during one example trial (subject S3). Traces are aligned to movement onset; period with partial slips and full sliding shadowed in grey.

A detailed description of the apparatus has been published previously [27]. In short, a transparent, horizontal glass plate was attached to two force/torque transducers (ATI nano 43, acquisition rate 1 kHz) and mounted horizontally on the end effector of an industrial robot (four-axis SCARA Denso HS-4535G). The subject's right index finger was placed in a support that ensured a precise guiding of the nail position and a constant angle between the long axis of the distal phalanx and the horizontal glass plate (approx. 20°; figure 1*a*). The normal force (*W*) applied to the fingertip was servo-controlled, whereas the tangential force (*F*) developed as a consequence of the controlled movements of the robot's end effector in the horizontal plane.

**Figure 2.**
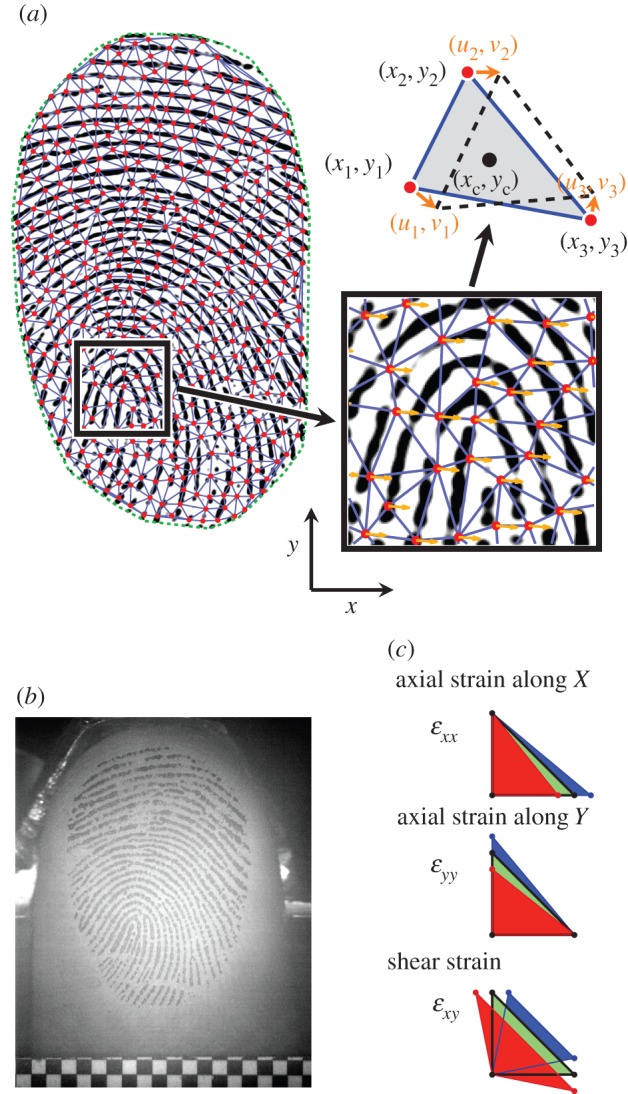
Computational procedures. (*a*) An image of the contact area. Feature points are superimposed in red, triangle edges in blue and optical flow vectors in orange (high-resolution cutout). For each triangle the calculated strain was attributed to its centre (*x*_c_,*y*_c_). (*b*) Example of original video image. (*c*) Schematic of each strain component. Relative to the original green triangle, red represents compressive strain or negative shear strain, whereas blue represents tensile strain or positive shear strain.

Images of the fingerpad were recorded through the glass plate by a camera (Mikrotron MC1362, resolution 1280 × 1024 pixels, up to 200 frames per seconds, fps) that was placed below the plate and had a clear view of its contact with the finger. A high contrast between fingerprint ridges and valleys was achieved by an optical arrangement which took advantage of the total internal reflection principle (figure 2*b*; [27,28]). The optics were adjusted to obtain a constant resolution of 52 pixels mm^−1^. A reference pattern printed on the glass plate was visible on each frame (bottom of figure 2*b*).

For each trial, the glass plate was first loaded on the fingertip at a given normal force, *W*. This load was kept as constant as possible during the whole trial through a closed-loop force controller (*W* standard deviation ranged 5.8–8.9% across subjects; figure 1*c*; [27]). The glass plate started to move tangentially 2 s after finger contact to minimize occlusion phenomena [29]. The plate moved in a given direction with a constant speed (except for an initial transient lasting less than 150 ms), for a total displacement of 14 mm. This distance ensured that the contact zone went from a fully stuck state to a fully developed slip state [27]. Following this displacement, the glass plate was then moved away from the fingertip. The frame rate was adjusted with the tangential speed to ensure 10 frames per millimetre (i.e. 50–200 fps for speeds 5–20 mm s^−1^).

**Figure 3.**
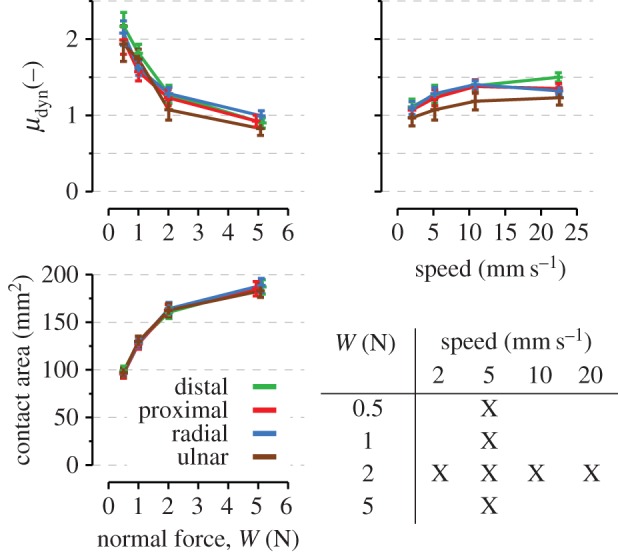
Coefficient of dynamic friction as a function of normal force (top left) and tangential speed (top right), and initial contact area as a function of normal force (bottom left). Colours represent directions as in figure 1*b*. Points and error bars are means and standard errors across subjects. Comparisons across normal forces were done for a fixed speed (5 mm s^−1^), and comparisons across speeds for a fixed normal force (*W* = 2N) as summarized in the bottom right table.

The experimental protocol was repeated five times in four directions (distal, proximal, radial and ulnar; figure 1*b*) and with varying normal force and speed (table inset, figure 3). In short, for each participant, 140 trials were performed (five repetitions × four directions × seven force per speed conditions), with experimental conditions applied in a randomized order within blocks of the same force condition.

### 2.2. Data analyses

#### 2.2.1. Force and position data

Force data were low-pass filtered with a fourth-order digital Butterworth filter with a cut-off frequency of 80 Hz and zero phase lag (the limiting factor was the image acquisition rate, i.e. 50 frames s^−1^). The glass plate displacement was determined from the reference frame displacement on the images (see ‘Displacement field’). The coefficient of dynamic friction, *μ*_dyn_, (equation (2.1)) was computed for each trial based on the ratio of the tangential force, *F*, to the normal force, *W*, once both had reached a plateau during total slippage.2.1
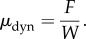


#### 2.2.2. Contact area

The apparent contact area (referred to simply as ‘contact area’ below) was obtained for each frame as previously described [27]. Briefly, the images were bandpass filtered in the spatial range of the fingerprint ridges spacing before applying mathematical morphology operations (greyscale closing and then opening) to merge together ridges and valleys. A threshold level was then computed using Otsu's method [30] to extract the contour of the contact zone from the resulting greyscale images. The convex hull enclosing the contact area was evaluated and then sampled with 50 equally spaced coordinate points.

#### 2.2.3. Displacement field

The displacement field was obtained using a computer vision technique called optical flow as described in [27]. A maximum number of features equally spaced by nine pixels were sampled in the initial contact area (figure 2*a*, red dots). The algorithm of Shi & Tomasi [31] was then used to select optimal features to track. These features were tracked from frame to frame with subpixel accuracy by applying Lucas & Kanade's algorithm [32,33] implemented with pyramidal refinement using 101 × 101 pixels subwindows in Matlab (Computer Vision Toolbox). Some features were removed or added during tracking depending on the contact area evolution, to obtain the *x*- and *y*-coordinates of up to 3000 features. To compute the median displacement vector of the contact plate, the tangential displacement of the reference pattern printed on the glass was evaluated using the same procedure as described above.

#### 2.2.4. Strain derivation

Green–Lagrange strains were estimated from the displacement gradient in the contact area by equation (2.2)2.2*a*
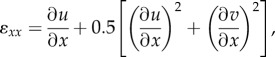
2.2*b*
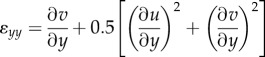
2.2*c*
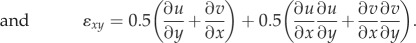
where *ɛ_xx_* and *ɛ_yy_* are the *axial strain* components aligned to the *x* and *y* axes, respectively, *ɛ_xy_* is the *shear strain*, and *u* and *v* are the *displacements* along *x* and *y* axes, respectively (figure 2*c*).

A Delaunay triangulation of the feature points sampled in the first frame was computed (figure 2*a*). For each triangle, displacement field gradients were derived (detailed description in electronic supplementary material) and attributed to the centre of the triangle. These gradients were used in equation (2.2) to compute the three strain rate components. We defined *strain rate* as the strain resulting from the displacement between two consecutive frames in the image sequence. The strain rates were estimated independently for each triangle and summed over time to obtain the *total strain* as a function of time, which is the actual deformation of the finger at a given instant relative to its initial state. In addition, the area of each triangle was computed.

#### 2.2.5. Principal strains

Principal strains were obtained by eigenvalue decomposition of the strain matrix *ɛ* (equation (2.3)). Because the strain matrix is symmetric, the eigenvectors ***υ***_1_ = (*υ_x_*_1_, *υ_y_*_1_) and ***υ***_2_ = (*υ_x_*_2_, *υ_y_*_2_) are orthogonal, i.e. ***υ***_1_·***υ***_2_ = 0. The eigenvalues *e*_1_ and *e*_2_ are the principal strain, and thus correspond to eigenvectors that are perpendicular in the *xy* plane. The principal strains *e*_1_ and *e*_2_ also correspond to the maximal tensile and compressive strains. The principal strain decomposition is thus equivalent to a rotation of the reference frame, so that the shear strain is cancelled and the axial strains take their maximal and minimal value2.3
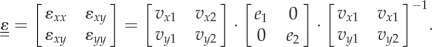


#### 2.2.6. Strain energy

A given stimulus applied to the fingertip transfers a certain amount of mechanical energy to the skin. This energy is transformed into deformations of the fingertip and into heat. The upper bound of the total energy transferred to the fingertip is the external work applied to the fingertip. An estimate of the total external work applied by the stimulus to the fingerpad (*U*_ext_) was evaluated by computing the integral of the tangential force along the displacement of the stimulus, from 0 to the displacement value reached when the tangential force was 90% of its plateau sliding value. This instant occurred just before the ‘steady-state slip’ [27].

By assuming an isotropic elastic material, it was also possible to compute the deformation energy specifically related to the observed surface strain. The strain energy density function, *u*_d_ (expressed in J m^−^³), can be written as a function of the strain components (*ɛ_xx_*, *ɛ_yy_* and *ɛ_xy_*; see equation (2.4)). We considered the cases of plane strain. The strain energy density was evaluated for each triangle in each frame, based on each total strain component. Young's modulus and Poisson's ratio values were chosen in the range of *in vivo* measurements, i.e. *E* = 1 MPa and *ν* = 0.4 [5,22].2.4
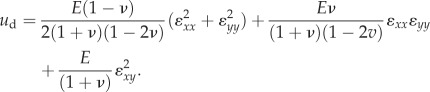


The total strain energy, *U* (mJ), was obtained by integrating the strain energy density over a given volume. As we did not measure the strains in the depth direction, the simplest estimate was to assume the surface strains to be uniform for a given depth (and the strains components related to the *z*-axis to be zero). Therefore, we integrated the strain energy *U* over the whole contact area and a depth corresponding to about half the distance between the bone and the stimulus during loading, i.e. 2 mm ([24]; equation (2.5)).2.5*a*
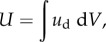
2.5*b*
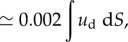
2.5*c*
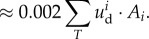


Finally, we computed the ratio of the total strain energy to the total external work (ratio = *U*/*U*_ext_), which is an estimate of the proportion of the stimulus energy actually deforming the skin at its contact surface. Again, this ratio was evaluated when the tangential force reached 90% of its plateau sliding value. Equations (2.4) and (2.5) show that the value of the strain energy *U* is directly proportional to both the Young modulus and the depth of integration. Those values were fixed in this study; future work might adjust these values to specifically match the fingers of different subjects.

#### 2.2.7. Strain normalization across subjects

Strains were experimentally obtained using unstructured meshes that differed across subjects. As the strain patterns were qualitatively and quantitatively similar across subjects, we obtained an average strain pattern, normalized across all subjects. We used a least-square procedure [34] to fit an ellipse on the 50 coordinate points of the contour of the contact area in the first frame of each trial. The geometric transformation (translation, scaling and rotation) was computed to fit this ellipse to a standard normalized ellipse and then applied to each triangle centre coordinate over the whole image sequence. Each strain component was then interpolated from the transformed mesh to a rectangular mesh defined on the standard ellipse. We computed the average value of each strain component across the five repetitions for each condition and for each individual subject (see electronic supplementary material, figure S1A). Then, the average values across all subjects were computed. Unless otherwise stated, the strain maps presented in this paper result from this averaging procedure.

#### 2.2.8. Fingerprint directional gradient field

The fingerprint directional gradient field (i.e. local fingerprint orientation) was evaluated based on the algorithm described in [35], for the first and the last image in each sequence. Briefly, gradients in *x*- and *y*-directions were obtained for each pixel and averaged over 32 × 32 pixels subwindows.

#### 2.2.9. Theoretical contact model

We compared our empirical data with that predicted by a Hertzian contact model between the finger and the glass, with the addition of friction as obtained by Cattaneo [36] and Mindlin [37], allowing partial slips. Even if Hertz contact assumptions are not perfectly met, this theory has been shown to predict contact properties (e.g. contact area) with good accuracy under normal and tangential loads [27]. The finger was modelled as an isotropic elastic sphere, and the glass plate was modelled as a rigid flat surface. Therefore, the contact area is a circle in this model. Using the Boussinesq–Cerruti equation, and based on the traction profiles obtained by [36] and [37] for partial slip of the contact area, we obtained the displacement field (*u,v*) across the contact area for a given tangential force (more details can be found in electronic supplementary material). This model is limited to the partial slip phase and does not predict strains during the full slip phase. The different parameters needed to compute the displacement field were extracted from data in this study (coefficient of friction, contact radius) or obtained from the literature (Young's modulus and Poisson's ratio; [5,22]). Displacement gradients were obtained numerically, and the strains were derived using equation (2.2). The strain energy density was estimated using equation (2.4).

### 2.3. Statistical analyses

All image processing, strain calculations and modelling were performed with Matlab (The MathWorks, Inc., USA). Some statistical analyses were computed with R (www.r-project.org). The influence of direction, force and velocity on the measured variables was analysed using repeated-measures ANOVAs. The five repetitions were averaged for every condition. Given the unbalanced design of the experiment (table inset, figure 3), separate ANOVAs were performed with direction × force and direction × velocity as factors. Sphericity was checked by Mauchly's test and, if needed, corrected with the Greenhouse–Geisser or Huynh–Feldt coefficients depending on epsilon. For directional data (figures 6 and 7), circular statistics were applied after doubling the angles and applying modulo 360° to obtain angles ranging 0–360°.

## 3. Results

Typical profiles for force, speed and stick ratio (SR; ratio of the stuck area to the total contact area) are presented in figure 1*c*. We defined a transient period (shaded in grey) that starts just after movement onset and ends when the tangential force plateaus. Strains were evaluated during this period.

As previously observed [25], the coefficient of friction varied across subjects but always followed the same trend, decreasing with the normal force (*p* < 0.001; repeated-measures ANOVA; figure 3) according to a power law (*μ* = *aW^b^* with *a* = 1.63 ± 0.31 and *b* = −0.33 ± 0.09; mean ± s.d.). The sliding speed (but not the direction) influenced the coefficient of friction (*p* = 0.002; superimposed coloured traces in figure 3, top panels). Friction increased with speed and reached a plateau level at approximately 10 mm s^−1^ with no significant differences between 10 and 20 mm s^−1^. The initial contact area varied with the normal force (*p* < 0.001).

### 3.1. Empirical strain patterns

**Figure 4.**
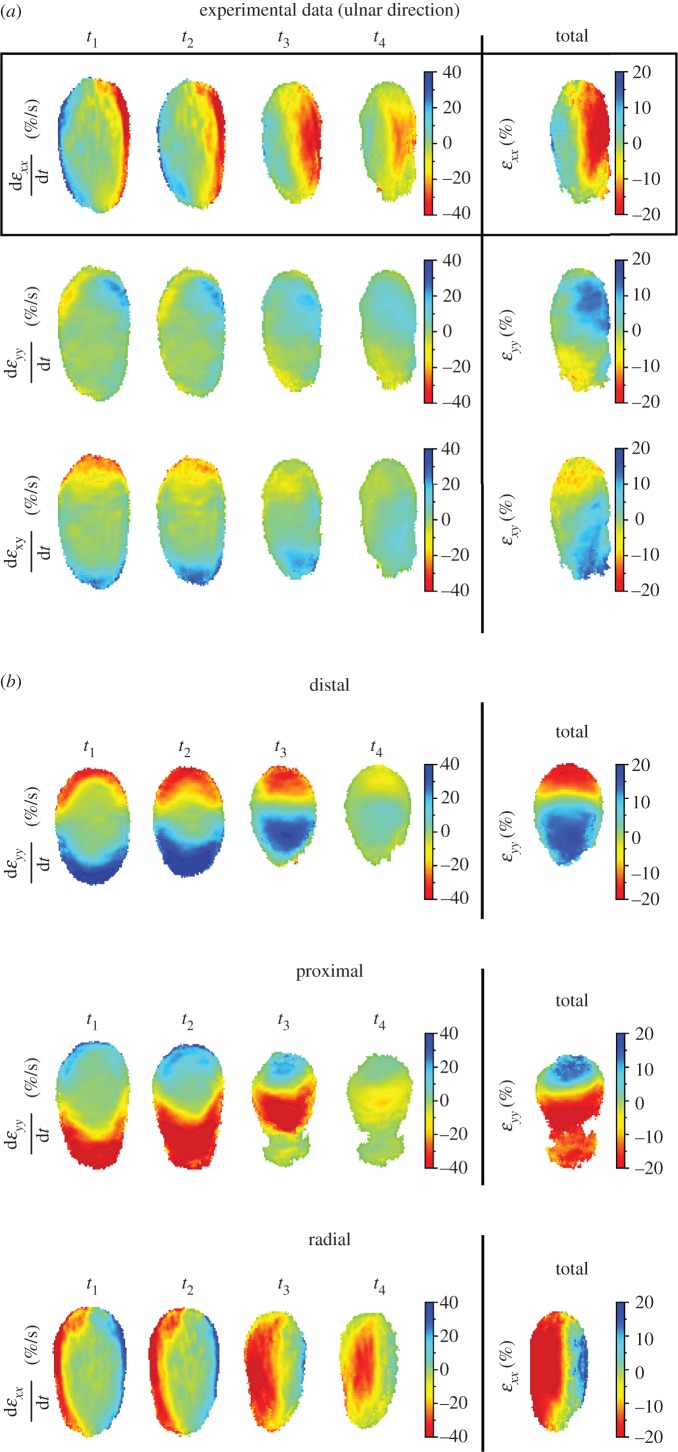
Strain evolution. Population average strain fields are represented as heat maps that show the evolution of strain fields in the contact area in the four directions: (*a*) each of the three rows represents a strain component. The component aligned to the movement (*ɛ_xx_*) is emphasized by the black box. (*b*) Each row represents the relevant strains given the movement directions; right column shows the total strains. Times *t*_1_–*t*_4_ are defined in figure 1*c*: *t*_1_ and *t*_2_ correspond to different instants before full slip, *t*_3_ corresponds to the instant of full slip and *t*_4_ is after full slip. Data from individual subjects are shown in electronic supplementary material, figure S1A.

Figure 4*a* presents normalized experimental data when the glass plate was translated in the ulnar direction (speed 5 mm s^−1^, 2 N normal force, data for the other directions with the same force and speed are shown in figure 4*b*). The illustrated measurements extend to the end of the transient period, when the strain rate had returned to zero and the fingerpad reached a homogeneous ‘steady-state slip’ [27]. Subject-specific traces are provided in electronic supplementary material, figure S1A.

A progressive strain wave was observed, propagating from the periphery to the centre of the contact area with the largest strain values located at the periphery of the contact. The strain wave was compressive ahead of the stuck area and tensile behind it. Depending on the stimulus direction, either the compressive part (ulnar, proximal and radial directions) or the tensile part (distal direction) was dominant (figure 4). The central undeformed zone was shifted distally in the distal and proximal cases, laterally in the radial case, and medially in the ulnar case (figure 4). These asymmetries were particularly apparent with large displacements (*t*_2_–*t*_4_) and less so at small displacements (*t*_1_). We also observed that skin at the border of the contact area lost contact with the glass plate in regions of high compressive strain during proximal translations.

### 3.2. Principal strains

**Figure 5.**
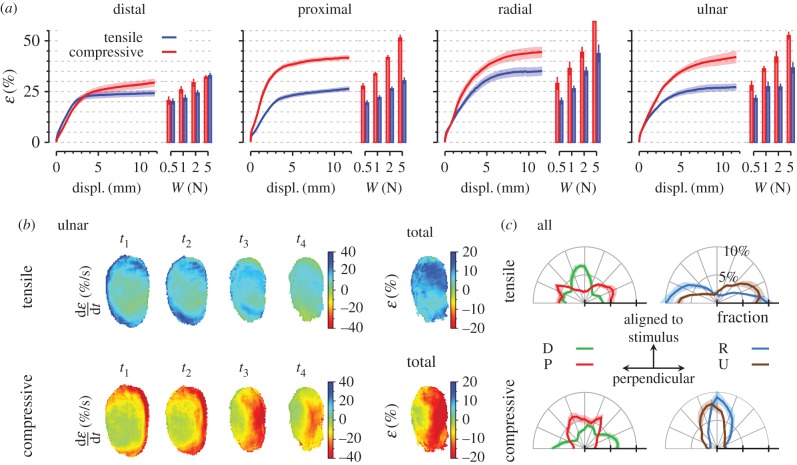
Principal strains. (*a*) Compressive and tensile strains for each direction, averaged across all subjects. Each left plot presents the evolution of maximal strain as a function of the tangential displacement (normal force = 2 N, speed = 5 mm s^−1^). Each right plot presents the final strain value at the end of the stimulation, for each normal force (*W*). (*b*) Heat maps of the evolution of the principal strain fields for a movement in the ulnar direction (averaged across all subjects). On the left, the evolution of the strain rates over time is presented in the four left columns (*t*_1_–*t*_4_ defined in figure 1*c*). In the right column, the total tensile and compressive strains are shown. (*c*) Polar histograms of the total principal compressive and tensile strain orientation across the contact area. The reference frame was rotated such that strains aligned to stimulus direction are aligned to vertical, while strains perpendicular are aligned to horizontal. Each bin covers 7.5°. Bin amplitude is expressed as a percentage of the total number of elements. Shaded areas and bars represent standard error. D is distal, P is proximal, R is radial and U is ulnar.

As explained in Methods, the principal strains that were computed from eigenvalue decomposition of the strain matrix represent directions without shear strain, i.e. with only compressive or tensile strains. Both the final tensile and compressive strain values depended on direction (*p* < 0.001; figure 5*a*). Strain amplitude increased with increasing normal force (*p* < 0.001) while speed did not affect the final strains. Compressive strain was higher than tensile strain in all directions but distal (*p* < 0.001; corrected Welch paired *t*-test). The peaks of both tensile and compressive strain rate were affected by speed (*p* < 0.001).

As observed previously, tensile and compressive strain profiles were symmetric for small deformations (*t*_1_), but asymmetric when plate displacement and fingertip deformation increased. Specifically, for ulnar stimulation, we observed compressive strain on the right lateral side of the contact area and tensile strain in the superior central part of the contact area (figure 5*b*).

Most of the compressive strains were aligned to the stimulus direction. Indeed, the distribution of compressive strains peaks along the stimulus direction (aligned vertically in figure 5*c*, bottom). On the other hand, most of the tensile strains were observed perpendicular to the stimulus direction in the radial and ulnar cases (aligned horizontally in figure 5*c*, top right). No obvious pattern was observed for the distal and proximal direction (figure 5*c*, top left).

### 3.3. Fingerprint effect

**Figure 6.**
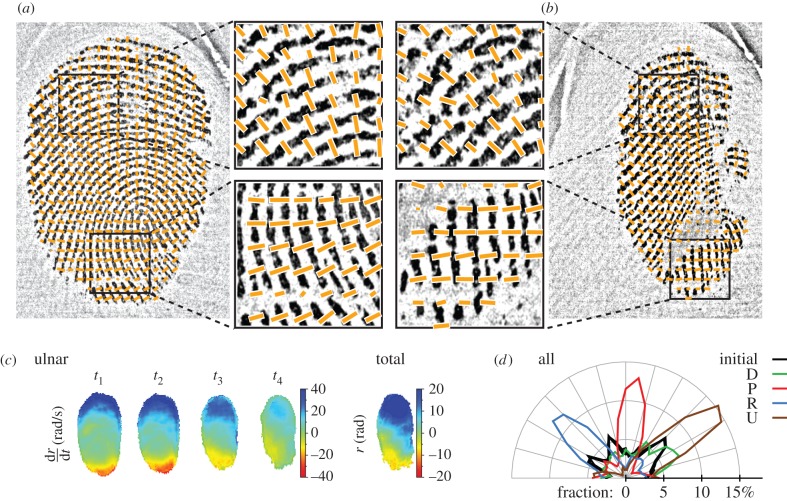
Fingerprint effect. (*a*) First and (*b*) last picture from a trial with plate movement in the ulnar direction from subject S3. Orange lines are superimposed and correspond to the estimated local gradient directions. (*c*) Heat maps of the evolution of the rotation field for the ulnar direction, averaged across subjects. Blue colour corresponds to anticlockwise rotations and red corresponds to clockwise rotations. (*d*) Polar histogram of the initial gradient orientation distribution (four traces superimposed in black) and the final orientation distribution for each direction (coloured traces), data from one single subject (S3). Each bin covers 7.5°. Bin amplitude is expressed as a percentage of the total number of elements. D is distal, P is proximal, R is radial and U is ulnar.

We observed significant torsion of the fingerprint ridges in the contact area as a consequence of tangential loading (figure 6). In short, the fingertip ridges tended to rotate, so that the fingertip's directional gradient field (orange lines in figure 6*a,b*) aligned to the stimulus direction. For instance, in response to ulnar stimulation, the distal part of the fingertip rotated anticlockwise, whereas the lower part rotated clockwise (figure 6*c*). Indeed, the differences between fingerprint gradient angle distribution at initial contact (traces in black) and final slipping state (in colour) were dramatically dependent on stimulus direction (figure 6*d*).

The four initial orientation distributions were the same for each stimulus direction and specific to a subject's fingerprint (superimposed in black in figure 6*d* for a given subject). The two-sample Kuiper's test was used to compare initial and final fingerprint orientation distributions. Of the 1120 recorded trials, 1099 showed a statistically significant difference in the orientation distribution (*p* < 0.05, not corrected for local correlations). Of the 21 non-significant pairs (1.9%), 17 were trials at the lowest normal force (and thus the smallest deformations). We observed that the histograms shifted and peaked towards the stimulation direction (coloured traces in figure 6*d*). This was verified by comparing the kurtosis of the direction distribution, which was higher at the final state compared with the initial state for all directions but distal.

### 3.4. Strain energy

**Figure 7.**
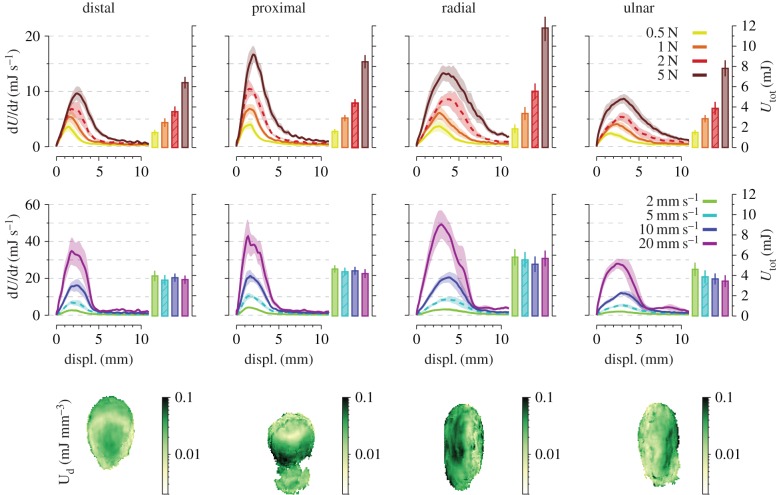
Strain energy evolution. The influence of normal force (top row) and speed (middle row) on the evolution of the total strain energy rate (d*U*/d*t*) with respect to stimulus displacement (left subplots) and final total strain energy (right subplots). Each column shows a different direction. Each trace and box shows the mean across subjects. Shaded areas and bars show standard error. Dashed traces and boxes represent the same condition in the top and the middle row. Comparison across normal forces was done for a fixed speed (5 mm s^−1^, vertical box), across speeds for a fixed normal force (2 N). The bottom row shows heat maps of the strain energy density distribution over the contact area in the final state. Data were averaged across subjects as described in Methods.

Figure 7 shows the evolution of the strain energy rate (d*U*/d*t*) over stimulus displacement, as well as the final strain energy (bar graphs). The cumulated energy increased monotonically until the end of the trial. The strain energy rate peaked close to *t*_3_ (figure 1*c*), i.e. the moment of transition to full slip (correlation: *r* = 0.74). The peak rate and total energy varied with the stimulus direction and increased with normal force (*p* < 0.01 in both cases). Additionally, the strain energy rate peaked later at higher forces (*p* < 0.001). Faster speeds resulted in increasing strain rate peaks (*p* < 0.001) that did not change the total amount of strain energy accumulated during the full trial.

The distribution of deformation energy stored in the tissues was markedly different depending on the stimulus direction (figure 7, bottom row), but also differed across subjects (electronic supplementary material, figure S1B).

Strain energy (*U*) was compared with the total external work performed on the fingertip (*U*_ext_). The median of ratio *U*/*U*_ext_ across all subjects was 0.50 (95% confidence interval: 0.36–0.65). Thus, given our assumptions—an elastic model of the fingertip with *E* = 1 MPa and *v* = 0.4, and a uniform displacement along the depth and limited to a layer of 2 mm—about half of the total external energy was used to mechanically deform the contact area during the stick to slip transition.

### 3.5. Model predictions

**Figure 8.**
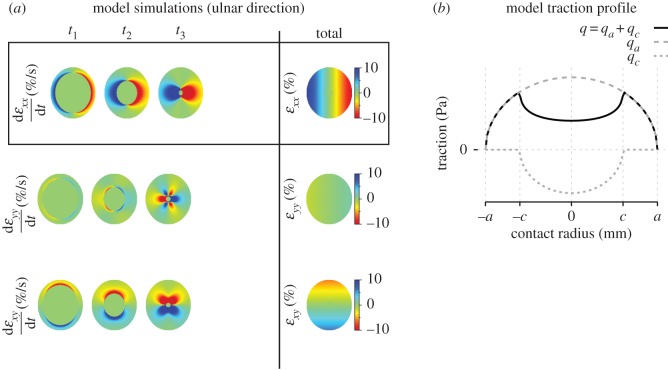
Model simulations. (*a*) Heat maps of simulated evolution of strain fields in the contact area for an ulnar movement. Each line represents a strain component. The left three columns show the strain rates over time (*t*_1_–*t*_3_ defined in figure 1*c*; the state at *t*_4_ undefined for the model). The right column shows the total strains. Red represents compressive strain or negative shear strain; blue represents tensile strain or positive shear strain. The component aligned to the movement (*ɛ_xx_*) is emphasized by the black box. *t*_1_ and *t*_2_ correspond to different instants before full slip. (*b*) Traction profiles predicted by equation 13 during the partial slip phase. For |*r*| < *c* (where *r* is the contact radius), contact area tissue is stuck. For *c* < |*r*| < *a*, contact area tissue is slipping.

Figure 8*a* shows the simulated evolution of the contact area strain in response to an ulnar stimulus, from the start of tangential loading to just before the instant of full slip (*t*_3_).

As expected, the model predicted strain waves propagating from the periphery to the centre, with the largest component being aligned to the stimulus direction. We observed qualitative similarities between the data and the model, most convincingly right after stimulus onset (i.e. for small strains). Indeed, the initial strain distributions (around *t*_1_) were in qualitative agreement with the data for all components. Those similarities did not hold for larger strain (*t*_2_−*t*_3_, final), where we observed three key differences between the model and the data. First, at the end of the transition from stick to slip, the simulation predicted that the fingertip would be deformed symmetrically across the contact area (excluding the centre), and that the highest strains would be observed at the contact area periphery. However, major asymmetries were observed in the experimental stimulus-aligned strain component, which showed not only a dominance of either compression or stretch, but also asymmetries in other components (e.g. comparing the second and third rows in figures 4*a* and 8*a*). Second, the model predicted the highest strain rates at a distance from the periphery, whereas the empirical data revealed that the highest strain rates actually and uniformly occurred at the very periphery of the fingerprint. Third, the model could not possibly capture the torsion effect described in the previous section and attributed to the fingerprint ridges.

## 4. Discussion

We measured the patterns of strain in fingertip skin as a smooth glass plate was slipped across its surface. We have observed how strain waves formed starting at the contact area periphery and moving towards the area's centre as less and less of the skin was in stable contact with the glass.

The strain amplitude and total energy density scaled with the normal force exerted on the finger up to 5 N. The range of force that we tested produced strains up to 50%. It was observed by Wang & Hayward [22] that under local traction, skin behaved almost linearly until a ‘knee’ value around 40–50% where the skin became much stiffer, a value that was likely not exceeded in this study. In addition, we did not observe any specific influence of tangential speed on the strains, as the strain rates simply scaled with speed. We concluded that the skin behaves elastically in the range of tested velocities [23]; fingertip viscoelasticity is therefore an unlikely explanation for the observed deviations from the theoretical model.

We also observed that amplitude and shape of the strain varied across directions, and found that this result could partly be attributed the fingerprint ridges. Indeed, local torsions were observed during traction, which tended to set the fingerprint perpendicular to the stimulus direction. The fingerprints play an important role in the mechanical properties of the fingertip skin. As observed by Wang & Hayward [22], the skin is locally stiffer along the fingerprints than across them. Take together, theirs and our observations imply that fingerprints shape how the finger is deformed during tangential traction. The role played by human fingerprints is not fully understood yet. For instance, it has been proposed that they increase friction and improve tactile discrimination capabilities. Fingerprints could shape or filter the vibrations elicited in the skin when scanning rough textures in a way that helps the nervous system to process them [38–40].

We quantified the proportion of mechanical energy that contributed to the observed strains to 50% (on average). This approximation should be taken with caution as it is directly dependent on two parameters taken from the literature (i.e. Young's modulus of 1 MPa and uniform deformation across 2 mm depth). Further investigation is needed to quantify how these parameters vary across subjects but also within subject trials (for instance as a function of the moisture level). Nevertheless, this study provides a reference value that can easily be adapted to different parameters and, accordingly, different finger properties. This study also demonstrates that the measured surface strains have a significant contribution to the overall deformation of the contact area that may be encoded by local tactile mechanoreceptors. Several previous studies showed that responses of type I afferents (SA-1 and FA-1) were closely related to strains within finger [1,2,16,17]. Therefore, it seems obvious that skin afferents will respond to the strain patterns documented in this study; as the partial slip increases, so will strain spread, intensity and energy. The afferent recruitment and firing rate should also increase, and the neural population response might thus provide information about the slip state of the finger.

To fully characterize the complete deformation of the skin, skin models must add components perpendicular to the stimulus surface. These components should include a compressive component perpendicular to the surface, global shearing of the fingerpad relative to fixed tissues (nail or bone), and surface strain outside the contact area. These components could contribute significantly to the total deformation energy, yet are much more complex to measure. Deformations also take place outside the contact area, particularly near the contact area border. These deformations invoke direction-selective responses in mechanoreceptors across the whole fingerpad, and thus convey relevant information about stimulus direction [24]. The extent to which remote tactile receptors could convey information about localized, transient finger-object slips remains to be demonstrated. Finally, this experiment was conducted with a flat glass surface, rather than a ‘natural’ surface like wood, suede or silk. Everyday ‘natural’ materials typically show larger values for static friction than dynamic friction, which is not the case for glass [27]. In these cases, Terekhov & Hayward [41] proposed that there exists a minimal adhesion surface area that suddenly slips once a tangential force threshold in reached. Nevertheless, even with natural surfaces, strains should appear at the border of the contact before overt slips occur, as presently observed.

An elastic model roughly predicted the compression and stretch region. However, it failed to explain the exact direction-dependent patterns of deformation observed. While the choice of elastic properties could be justified, the substantial deviations observed are most likely explained by the deviation from two assumptions of the model: (i) homogeneity and isotropy (as shown by the specific effect of the fingerprints) and (ii) spherical geometry. First, the specific local geometry, the layout of the skin layers and underlying tissues and the presence phalange bone most likely affects the observed strain patterns. Second, the finger is clearly not spherical. In short, while Hertzian contact theory and linear elasticity qualitatively explain first-order phenomena, they fail to explain the complex strain patterns observed in our data.

The two low-threshold, fast-conducting afferents most closely linked to the detection of local frictional slips in humans have small receptive fields and are called type FA-1 and SA-1, i.e. fast and slowly adapting afferents, respectively [42]. FA-1 afferents comprise about 50% of all from human fingertips, whereas SA-1 constitute about 25% [43]. They both respond promptly to slips in or close to their receptive fields (i.e. to ‘localized slips'), whereas type SA-1 afferents in addition encode local static strain patterns. Type SA-1 responds poorly to vibratory frequencies above 10 Hz, whereas type FA-1 is most sensitive to vibrations with frequencies between 5 and 50 Hz, i.e. close to the upper range of frequencies captured by our video recordings (50 Hz). In contrast, Pacinian corpuscles (or type FA-2) that comprise less than 10% of the afferents from the human fingertip show their highest sensitivity to 200–300 Hz vibrations, i.e. events far beyond what we have captured in this study. However, experimental evidence and their locations in the tissues suggest that type FAII is primarily important for encoding global mechanical transients. It thus seems reasonable to claim that we in this study have captured events of direct relevance for the large majority of the low-threshold afferents in the human fingertip involved in encoding localized slip.

### 4.1. Perspectives

In addition to motivating further research on the neurophysiological encoding of mechanical states and events in the finger, this work has implications for the design of haptic interfaces and tactile displays. Indeed, tactile interfaces can produce tangential skin traction [44]. Given our results, it seems possible that a pattern of traction such that one side of the finger is compressed, and the other side is stretched may produce the sensation of a tangential force component or even a slip in the absence of actual tangential force.

## Supplementary Material

Supplementary methods and figures
