# Supplementary material for: Surface strain measurements of fingertip skin under shearing
Source: J R Soc Interface. 2016 Feb;13(115):20150874. doi: 10.1098/rsif.2015.0874 (PMC4780562; doi:10.1098/rsif.2015.0874)
Supplement: Supplementary methods and figures [file rsif20150874supp1.doc]

# Supplementary methods

## Displacement gradient computation

For each triangle, the vertices coordinates (*x_1_,y_1_*), (*x_2_,y_2_*) and (*x_3_,y_3_*) and the displacement vectors at these coordinates (*u_1_,v_1_*), (*u_2_,v_2_*) and(*u_3_,v_3_*) are known (see Figure 2a). Here, we only describe the derivation for the field *u*, but the same can be done for the field *v*. In the 3D space (*x,y,u*), the plane passing through (*x_1_,y_1_,u_1_*), (*x_2_,y_2_,u_2_*) and (*x_3_,y_3_,u_3_*) is of the form described by equation 6, where the scalar coefficients *A* and *B* are also the gradient of field *u*: A=du/dx, B=du/dy and the coefficient *C* is a translation constant.


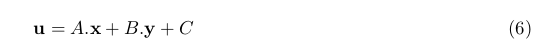


The three coefficients can be obtained by solving a 3x3 linear system (equation 7).


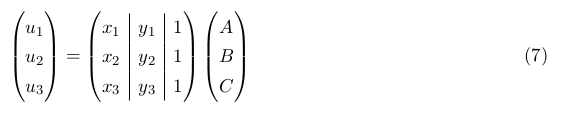


As we are only interested in computing the gradient of the displacement field *u*, we build a 2x2 linear system (equation 8) where C is removed by computing differences between lines in equation 7. This system is solved to obtain the gradient. The obtained gradient is attributed to the center of the triangle (*x_c_,y_c_*) (see Fig. 2a).


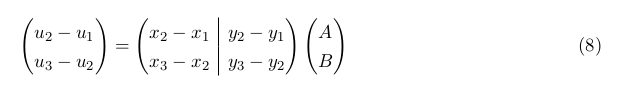


## Model equations

Displacements are obtained from Boussinesq and Cerruti integrals (equation 9), for a given traction profile.


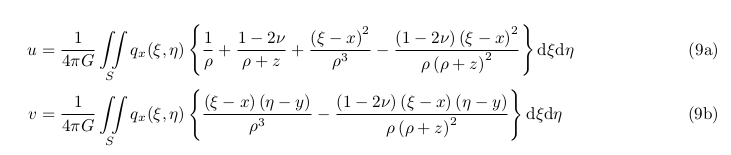


with . *G* is the shearing modulus, and ν is the Poisson's ratio.

If we focus on displacements evaluated on the contact surface, z=0 and Equation 9 reduces to Equation 10.


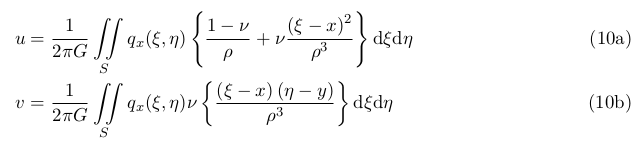


with .

The integration of equations 10 was done for a circular traction region of radius *a* with a profile given by Equation 11.


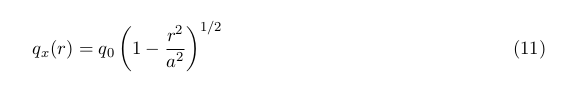


It leads to expressions for displacement component aligned to the traction, *u*, and perpendicular to the traction, *v*, inside and outside the circular region (equation 12) as shown in [45–47].


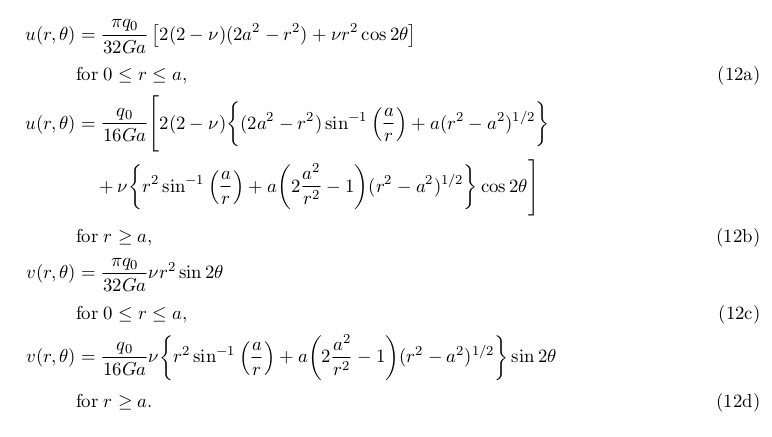


A traction distribution profile during the stick to slip evolution has been proposed by [36] and [37]. It was proposed that the total traction profile *q* is well approximated by the sum of two traction profile: (i) A traction profile in the whole contact area at the point of slip *q_a_* and (ii) the traction profile in the stuck region *q_c_*, see Equation 13. The profile of q_c_ is presented in fig 8b. The total displacements were obtained by summing the displacement produced by these two traction profiles.


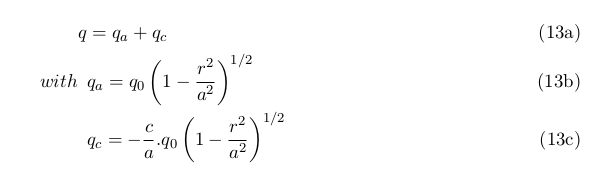


## Supplementary figure A

Heat maps of the evolution of strain fields along the stimulus direction. Data is shown for all four directions and for each of the 8 subjects (one subject per row). Layout as in Fig.4 and t_1_–t_4_ defined in Fig. 1c.

### Supplementary figure B

Heat maps of the final strain energy density distribution for the 8 subjects (one subject per row).

References

44. Johnson KL. 1955 Surface interaction between elastically loaded bodies under tangential forces. *Proc. R. Soc. Lond. A* **230**, 531–548. (doi:[10.1098/rspa.1955.0149](http://dx.doi.org/10.1098/rspa.1955.0149))

45. Johnson KL. 1985 *Contact mechanics*. Cambridge, UK: Cambridge University Press.

46. Jäger J. 1993 Elastic contact of equal spheres under oblique forces. *Arch. Appl. Mech*. **63**, 402–412. (doi:[10.1007/BF00805740](http://dx.doi.org/10.1007/BF00805740))
